# Properties of BK-type Ca^+^^+^-dependent K^+^ channel currents in medial prefrontal cortex pyramidal neurons in rats of different ages

**DOI:** 10.3389/fncel.2013.00185

**Published:** 2013-10-25

**Authors:** Aneta Książek, Wioletta Ładno, Bartłomiej Szulczyk, Katarzyna Grzelka, Paweł Szulczyk

**Affiliations:** ^1^Department of Physiology and Pathophysiology, Medical University of WarsawWarsaw, Poland; ^2^Department of Drug Technology and Pharmaceutical Biotechnology, Medical University of WarsawWarsaw, Poland

**Keywords:** BK single-channel currents, BK whole-cell currents, action potentials, paxilline, age-dependent properties

## Abstract

The medial prefrontal cortex (PFC) is involved in cognitive functions, which undergo profound changes during adolescence. This alteration of the PFC function derives from neuron activity, which, in turn, may depend on age-dependent properties and the expression of neuronal ion channels. BK-type channels are involved in controlling both the Ca^+^^+^ ion concentration in the cell interior and cell excitability. The purpose of this study was to test the properties of BK currents in the medial PFC pyramidal neurons of young (18- to 22-day-old), adolescent (38- to 42-day-old), and adult (60- to 65-day-old) rats. Whole-cell currents evoked by depolarizing voltage steps were recorded from dispersed medial PFC pyramidal neurons. A selective BK channel blocker – paxilline (10 μM) – irreversibly decreased the non-inactivating K^+^ current in neurons that were isolated from the young and adult rats. This current was not significantly affected by paxilline in the neurons obtained from adolescent rats. The properties of single-channel K^+^ currents were recorded from the soma of dispersed medial PFC pyramidal neurons in the cell-attached configuration. Of the K^+^ channel currents that were recorded, ~90% were BK and leak channel currents. The BK-type channel currents were dependent on the Ca^+^^+^ concentration and the voltage and were inhibited by paxilline. The biophysical properties of the BK channel currents did not differ among the pyramidal neurons isolated from young, adolescent, and adult rats. Among all of the recorded K^+^ channel currents, 38.9, 12.7, and 21.1% were BK-type channel currents in the neurons isolated from the young, adolescent, and adult rats, respectively. Furthermore, application of paxilline effectively prolonged the half-width of the action potential in pyramidal neurons in slices isolated from young and adult rats but not in neurons isolated from adolescent rats. We conclude that the availability of BK channel currents decreases in medial PFC pyramidal neurons of adolescent rats compared with those in the neurons of young and adult rats while their properties did not change across ages.

## INTRODUCTION

The medial prefrontal cortex (PFC) is involved in cognitive functions, such as decision making (Sul et al., 2010), reasoning (Coricelli and Nagel, 2009), and planning (Barbey et al., 2009), and these functions undergo profound changes during adolescence (Selemon, 2013). Dysfunction of the PFC has been implicated in many neuropsychiatric disorders including schizophrenia (Williams and Castner, 2006), depression (Lemogne et al., 2009), and drug dependence (Huang et al., 2007). These illnesses are age-dependent because they usually begin during adolescence (Spear, 2000; McCutcheon and Marinelli, 2009).

It is expected that changes in PFC cognitive function or dysfunction during adolescence derive from altered PFC neuron activity, which, in turn, depends on the properties and expression of ion channels and/or on communication between neurons. Indeed, during adolescence, PFC pyramidal neurons undergo structural and functional transformations (Zhu, 2000; Andersen, 2003); for example, in mature rats, L-type Ca^+^^+^ channel function and PKA signaling increase in pyramidal neurons compared with that in young animals (Heng et al., 2011). Additionally, the action potential amplitude increases during development from youth to adolescence, most likely due to a Na^+^ current density increase (Zhang, 2004). The densities of voltage-dependent K^+^ currents increase during the postnatal period and reach a plateau in adolescence (Guan et al., 2011). Moreover, the expression of D_1_ receptors increases (Brenhouse et al., 2008) in adolescents compared with that in young rats in the medial PFC pyramidal neurons. Furthermore, the properties of medial PFC pyramidal interneurons change during adolescence; for example, there are more fast-spiking interneurons with Ca^+^^+^-permeable AMPA receptors (Wang and Gao, 2010) and a decrease in the number of fast-spiking interneurons with NMDA receptors (Wang and Gao, 2009). There is also an increase in the number of PFC interneurons responding to D_2_ receptor stimulation in adolescent rats (Tseng and O’Donnell, 2007). It is assumed that major structural and functional changes in developing PFC pyramidal neurons and interneurons occur during adolescence and that most of these changes are complete in adult animals.

Ca^+^^+^-dependent K^+^ BK-type channels, which conduct an outward K^+^ current, are widely expressed in the neocortex, including medial PFC pyramidal neurons (Benhassine and Berger, 2005). BK channels are activated during an increase in the Ca^+^^+^ ion concentration in the cytoplasm. The Ca^+^^+^ ion concentration predominantly increases due to the opening of voltage-dependent Ca^+^^+^ channels during the firing of action potentials. BK channels are elements of a negative feedback mechanism that contributes to action potential repolarization, which results in the closing of voltage-gated Ca^+^^+^ channels and in the restoration of the resting membrane potential (Sah and Faber, 2002; Benhassine and Berger, 2005). Therefore, BK channels control the inward Ca^+^^+^ current and the intracellular concentration of Ca^+^^+^ ions.

BK-type Ca^+^^+^-dependent K^+^ channel currents have been studied during the prenatal and early days of postnatal development. The density of BK channels increased during the first postnatal days in the neocortical pyramidal neurons of the sensorimotor cortex (Kang et al., 2000), in the cerebellum (Muller et al., 1998) and in the calyx of Held presynaptic terminals (Nakamura and Takahashi, 2007).

The purpose of this study was to compare the availability and properties of Ca^+^^+^-dependent K^+^ BK-type channels in medial PFC pyramidal neurons in young, adolescent, and adult rats.

## MATERIALS AND METHODS

The experimental procedures that were used in this study were approved by the Second Local Ethical Committee. Experiments were performed on 18- to 22-day-old (young), 38- to 42-day-old (adolescent), and 60- to 65-day-old (adult; Spear, 2000) Wistar rats (WAG Cmd) that had been obtained from a local animal house.

### PREPARATION OF SLICES

Slices were prepared as described previously (Witkowski and Szulczyk, 2006; Witkowski et al., 2008, 2012; Szulczyk et al., 2012). The animals were decapitated under ethyl chloride anesthesia, after which their brains were exposed, removed, and placed in a cold (1–4°C), oxygenated, extracellular solution containing the following (in millimolar): sucrose (234), KCl (2.5), NaH_2_PO_4_ (1), glucose (11), MgSO_4_ (4), HEPES-Cl [N-(2-hydroxyethyl)piperazine-N′-(2-ethanesulphonic acid)] (15), CaCl_2_ (0.1), and ascorbic acid (1). The pH of the solution was adjusted to 7.4 with *N*-methyl-D-glucamine (NMDG), and its osmolality was 330 mOsm/kg H_2_O. Coronal slices of cerebral prefrontal tissue with thicknesses of either 300 or 400 μm were prepared using a vibratome (Vibratome 1000, Pelco International, CA, or Leica VT1200S, Germany).

Prior to recording, the slices were stored at room temperature (21–22°C) in a solution containing the following (in millimolar): NaCl (118), KCl (2.5), CaCl_2_ (0.5), MgSO_4_ (3), NaHCO_3_ (25), NaH_2_PO_4_ (1.25), and glutathione (0.1). The solution was bubbled with a mixture of 95% O_2_ and 5% CO_2_ (the pH was 7.4, and the osmolality was 310 mOsm/kg H_2_O). All recordings were performed at room temperature (21–22°C).

### PREPARATION OF DISPERSED PYRAMIDAL NEURONS

Sections of slices (2.2–3.5 mm anterior to Bregma, 3–5 mm below the upper cortical surface, and 0.6–0.9 mm from the midline; Kolb, 1984; Berger et al., 1991; öngür and Price, 2000) were dissected and transferred to a solution that was bubbled with O_2_ and that contained the following compounds (in millimolar): NaCl (135), HEPES-Cl (10), KCl (5), MgSO_4_ (1), CaCl_2_ (0.1), and glucose (10), and 1 mg/ml protease type XIV (Sigma-Aldrich, Poland). The pH of the solution was adjusted with NaOH to 7.4, and the osmolality was 300 mOsm/kg H_2_O. Enzymatic action was allowed to occur for 15–30 min at 32°C, after which it was arrested by replacing the solution that was bathing the slices three times with an identical solution that lacked proteases. Parts of the slices were mechanically dispersed using Pasteur pipettes. The dispersed neurons were then transferred to a recording chamber (type RC-24E, Warner Instr., USA) and placed on the stage of an inverted microscope (Nikon Instech Co., Ltd., Kawasaki, Kanagawa, or Olympus Corporation IX2, Japan). Individual cells were identified using Hoffman or DIC optics (magnification 400×). Surface pyramidal neurons that possessed the following characteristics were selected for recording: a triangular shape; a smooth, three-dimensional appearance; residual apical and basal dendrites; and a short axon at its base (as in **Figure 1B** in Witkowski and Szulczyk, 2006).

**FIGURE 1:**
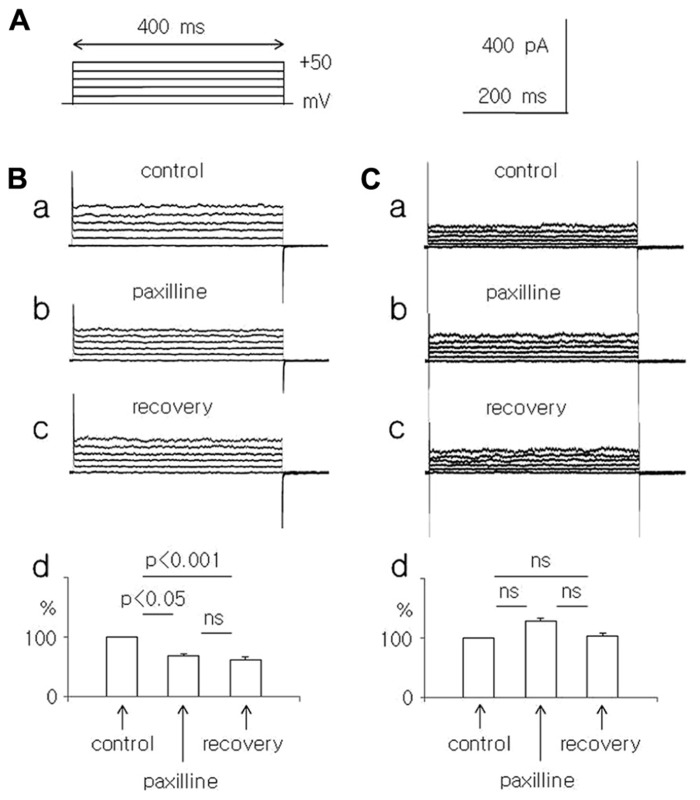
**Effect of paxilline (10 μM) on the whole-cell currents recorded in dispersed pyramidal neurons isolated from young and adolescent rats.** **(A)** Voltage protocol applied to evoke a whole-cell current. Rectangular voltage steps lasting 400 ms were applied in 10 mV increments from 0 to +50 mV, once every 4 s. **(B)** Whole-cell currents recorded before (a, control), at the end of a 5-min paxilline application (b, paxilline) and after a 20-min paxilline wash-out (c, recovery) in pyramidal neurons obtained from young rats. Average relative amplitudes of the whole-cell current (vertical axis) evoked by +50 mV voltage steps before (control), during paxilline application (paxilline), and after paxilline wash-out (recovery) in pyramidal neurons obtained from young rats (d). **(C)** Whole-cell currents recorded before (a, control), at the end of a 5-min paxilline application (b, paxilline), and after a 20-min paxilline wash-out (c, recovery) in pyramidal neurons obtained from adolescent rats. Average relative amplitudes of the whole-cell current (vertical axis) evoked by +50 mV voltage steps before (control), during paxilline application (paxilline), and after paxilline wash-out (recovery) in pyramidal neurons obtained from adolescent rats (d).

### CURRENT RECORDINGS IN THE WHOLE-CELL CONFIGURATION FROM DISPERSED PYRAMIDAL NEURONS

The pipette solution for current recordings in the whole-cell configuration contained the following (in millimolar): K acetate (110), KCl (35), HEPES-Cl (10), CaCl_2_ (0.01), MgATP (4), Na_2_GTP (0.3), and Na_2_-phosphocreatine (10). The pH of the solution was adjusted to 7.3 using KOH, and the osmolality was adjusted to 300 mOsm/kg H_2_O using sucrose. The cells were continuously perfused with an external solution that was delivered to the whole bath at a rate of 2 ml/min. This solution contained the following (in millimolar): KCl (145), CaCl_2_ (2), MgCl_2_ (2), glucose (10), HEPES-Cl (10), LaCl_3_ (0.005) or CdCl_2_ (0.1), and TTX (tetrodotoxin citrate, 0.0005). The pH of the solution was adjusted to 7.4 using KOH, and the osmolality was adjusted to 330 mOsm/kg H_2_O using sucrose.

Currents were recorded using an Axopatch 1D amplifier. pClamp software was used (Axon Instruments and Molecular Devices, USA). Pipettes (including the pipettes used in this study for channel current recordings and membrane potential recordings) were fabricated from borosilicate glass capillaries (O.D. 1.5 mm, I.D. 0.86 mm; Harvard Apparatus, Edenbridge, UK) using a P-87 puller (Sutter Instruments, Inc., Novato, CA, USA) and were then fire-polished. The pipette tips were coated with Sylguard. The junction potential was nulled with the pipette tip immersed in the bath. After gigaseal formation, the electrode capacitance was compensated by the circuit of the amplifier. The membrane was ruptured spontaneously or by suction. The access resistance ranged from 5 to 7 MΩ. A series resistance compensation of 80% was routinely applied. Currents were digitized at 10 kHz and filtered using an amplifier with a pole Bessel filter (2 kHz).

### CHANNEL CURRENT RECORDINGS IN THE CELL-ATTACHED CONFIGURATION FROM DISPERSED PYRAMIDAL NEURONS

The pipette solution for single-channel current recordings contained the following (in millimolar): K acetate (130), HEPES-Cl (10), MgCl_2_ (2) and CaCl_2_ (2), and TTX (0.001). The pH of the solution was 7.3 and was adjusted using NMDG, and the osmolality was adjusted to 305–310 mOsm/kg H_2_O (with sucrose). The extracellular solutions that were delivered to the recording chamber contained the same ingredients with the following exceptions: K acetate was replaced by KCl, and either LaCl_3_ (0.005) or CdCl_2_ (0.1) was included. The osmolality was adjusted to 330–335 mOsm/kg H_2_O.

The pipette open-tip resistance in the bath was 7.5–10 MΩ. The junction potential was nulled with the pipette tip immersed in the bath. Channel currents were recorded after gigaseal formation. All potentials were expressed in terms of the cytoplasmic side of the patch membrane. Data were digitized at 20 kHz, filtered with a four-pole low-pass Bessel filter (2 kHz) and stored on a computer. The channel current traces were smoothed using pClamp 9.0 software. The single-channel conductance was calculated as the slope of the best-fit line to the linear range of the I-V plot for that channel using the following equation: Q = I/V, where Q is the channel conductance, I is the maximum amplitude of the channel current, and V is the membrane potential. The probability of a channel being open (*P*o) was calculated as follows: *P*o = *t*_open_/*t*_total_, where *t*_open_ is the total open time of the channel, and *t*_total_ is the total analysis time.

### MEMBRANE POTENTIAL RECORDINGS

Prior to the membrane and action potential recordings, the slices were incubated for 40 min in a warm (34°C) extracellular solution containing the following (in millimolar): NaCl (130), KCl (2.5), glucose (10), NaHCO_3_ (25), NaH_2_PO_4_ (1.25), MgCl_2_ (2), and CaCl_2_ (2). The solution was bubbled with a mixture of 95% O_2_ and 5% CO_2_ at a pH of 7.4 and an osmolality of 320 mOsm/kg H_2_O. Membrane potentials were recorded in the same solution, which also included blockers of GABAergic and glutaminergic transmission (50 μM picrotoxin, 10 μM DNQX, and 50 μM AP-4). The pipette solution contained the following at a pH of 7.25 and an osmolality of 280 mOsm/kg H_2_O (in millimolar): potassium gluconate (105), KCl (20), HEPES-Na^+^ (10), ATP (4), MgCl2 (4), Na_2_GTP (0.3), and Na_2_-phosphocreatine (10).

The slices were placed in a bath chamber (RC-24E, Warner Instruments, LLC, MA, USA) on the stage of an upright Nikon microscope (Eclipse E600FN; Nikon Instech Co., Ltd., Japan). The neurons were visualized using infrared differential interference contrast with a 40× water immersion objective, a camera (C7500-50) and a camera controller (C2741-62) from Hamamatsu Photonics K.K (Japan). After obtaining a gigaseal, the membrane was ruptured (access resistance 5–7 MΩ). The recordings were performed in current-clamp configuration from layer V pyramidal neurons of the infralimbic and prelimbic medial PFC at a depth of 600–800 μm from the cortical surface. pClamp 9.0 software for MultiClamp 700A and a Digidata 1332A were used (Molecular Devices, CA, USA). The membrane potential recordings were digitized at 20 kHz and filtered at 2 kHz.

### CHEMICAL COMPOUND DELIVERY

The neurons in the slices or the dispersed neurons were continuously superfused with artificial extracellular solution delivered to the entire recording chamber. In addition, the tested dispersed neuron was washed out using the extracellular solution that flowed from the tubing (inside diameter 250 μm, EVH-9, Bio-Logic Science Instruments, France). Its tip was placed next to the tested cell with the use of an independent micromanipulator. The solution flowing from the tubing was identical to the solution that was delivered to the entire bath chamber and could also contain a different concentration of Ca^+^^+^ ions: 0 mM of Ca^+^^+^ with 0.5 mM of EGTA (ethylene glycol tetraacetic acid), 0.1 μM of Ca^+^^+^, 10 μM of Ca^+^^+^, paxilline (10 μM, Tocris UK), or TEA-Cl (1.5 mM, tetraethylammonium chloride, Sigma-Aldrich). Paxilline was dissolved in dimethyl sulfoxide (DMSO, the final concentration of DMSO was 0.01%). When the effect of paxilline was tested, the control solution flowing from the tubing also contained DMSO at a concentration of 0.01%.

When the effect of paxilline (10 μM) was tested on the pyramidal neurons in slices, paxilline was delivered to the entire bath chamber and the control solution that was delivered to the chamber also contained DMSO in a concentration of 0.01%.

The chemical compounds that were used were purchased from Sigma-Aldrich, Tocris (UK), Latoxan (France), or Polskie Odczynniki Chemiczne (Poland).

### STATISTICS

All of the results presented in this paper are shown as the means ± SEM. GraphPad InStat software v3.06 (GraphPad Software, Inc., CA, USA) was used for the statistical analyses.

## RESULTS

### EFFECT OF PAXILLINE ON NON-INACTIVATING WHOLE-CELL CURRENTS IN DISPERSED MEDIAL PFC PYRAMIDAL NEURONS IN YOUNG, ADOLESCENT AND ADULT RATS

The effects of paxilline (10 μM) and TEA-Cl (1.5 mM) were tested on total membrane K^+^ currents recorded in the whole-cell configuration from dispersed medial PFC pyramidal neurons. Na^+^ ions were absent from the intracellular (pipette) and extracellular solutions. The extracellular solution contained Na^+^ and Ca^+^^+^ channel blockers and a high concentration of K^+^ ions to record the whole-cell membrane currents in “symmetrical” K^+^ solutions. The concentration of Ca^+^^+^ ions in the pipette (intracellular) solution was 10 μM. The K^+^ currents were evoked by rectangular voltage steps from 0 to +50 mV in 10 mV increments lasting 400 ms and applied once every 4 s (**Figure 1A**).

Current run-down was observed immediately after gaining cell access; we recorded a decrease in the maximum K^+^ current amplitude evoked by the same voltage steps. To avoid the effects of current run-down on the experimental data, the K^+^ currents were recorded once every 2 min until their amplitude was unchanged for consecutive current recordings. When the current amplitude stabilized, the experimental data were collected.

Paxilline – an intracellular blocker of BK-type Ca^+^^+^-dependent K^+^ currents – was applied extracellularly. Because paxilline is lipophilic (Knaus et al., 1994), it can enter the cytoplasm and effectively block BK channels (Holm et al., 1997; Li and Cheung, 1999; Faber and Sah, 2003; Benhassine and Berger, 2009). Current amplitudes were measured as an average current over 100 ms in the middle of the voltage step before, after 5 min of paxilline application and 20 min after paxilline washout.

In the pyramidal neurons of the young rats, the outward K^+^ current, which was evoked by a +50 mV voltage step, decreased significantly to 67.0 ± 4.7% during the paxilline application and to 61.2 ± 5.2% during washout, compared with the control current (100%, *n* = 8, Friedman test followed by Dunn’s test, Fr = 12.97, *p* = 0.0015, **Figures 1Ba–d**).

In the pyramidal neurons isolated from the adolescent rats, the outward current, which was evoked by a +50 mV voltage step, did not significantly change during the paxilline application (128.1 ± 9.7% of the control) and during the recovery (103.4 ± 8.5%) compared with the control current of 100% (*n* = 8, Friedman test followed by Dunn’s test, Fr = 3.16, *p* = 0.206, **Figures 1Ca–d**).

**FIGURE 2:**
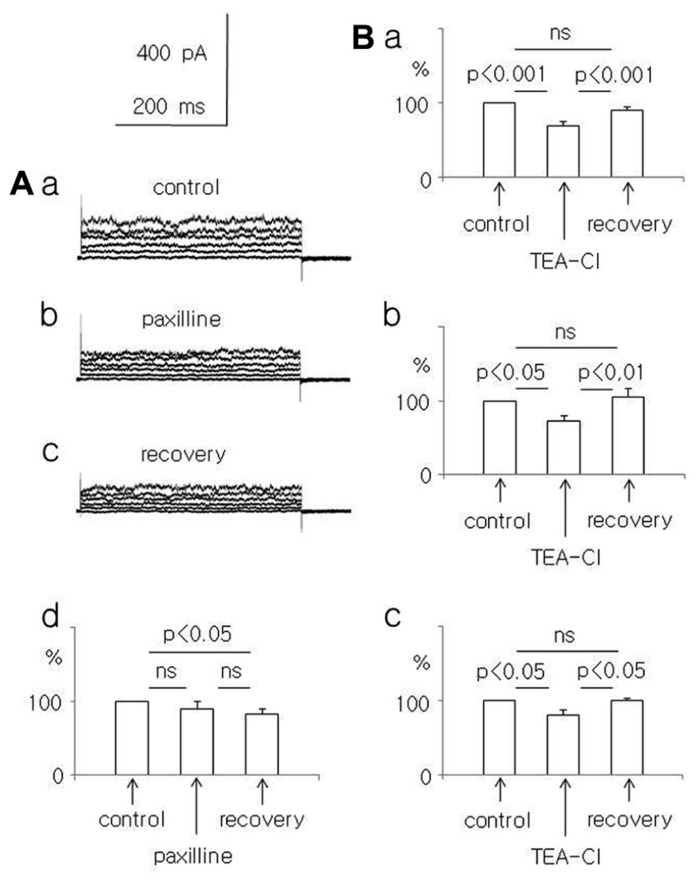
**Effect of paxilline (10 μM) and TEA-Cl (1.5 mM) on the whole-cell currents recorded from dispersed pyramidal neurons.** **(A)** Whole-cell currents recorded before (a, control), at the end of a 5-min paxilline application (b, paxilline), and after a 20-min paxilline wash-out (c, recovery) in neurons isolated from adult rats. Averaged relative amplitudes (vertical axis) of the whole-cell currents evoked by +50 mV voltage steps before (control), during paxilline application (paxilline), and after paxilline wash-out (recovery, d). **(B)** Average relative amplitudes of the whole-cell current (vertical axis) evoked by +50 mV voltage steps before (control), at the end of a 5-min TEA-Cl application (TEA-Cl), and after a TEA-Cl wash-out (recovery) in pyramidal neurons obtained from young (a), adolescent (b), and adult (c) rats.

In adult rat pyramidal neurons, the current amplitude evoked by +50 mV voltage steps decreased during paxilline application and after washout. Currents were 90.1 ± 9.3 and 82.4 ± 7.7%, respectively, compared to the baseline current (100%). The decrease in current amplitude was significant only during wash-out (Friedman test followed by Dunn’s test, Fr = 6.9, *p* = 0.031, *n* = 8, **Figures 2Aa–d**).

Additional experiments were performed to exclude the possibility that run-down caused the current amplitude decrease observed in the paxilline-treated neurons of young and adult rats. We tested the effect of the unspecific and reversible K^+^ channel current blocker TEA-Cl (1.5 mM) on the whole-cell BK current. We found that TEA-Cl reversibly decreased the current amplitude in neurons isolated from young, adolescent, and adult rats. In neurons isolated from young rats, the current amplitude decreased to 69.8 ± 4.4% during TEA-Cl application (*p* < 0.001) and recovered to 91.1 ± 3.9% after washout (*n* = 11, an ANOVA *F*_(2,20)_ = 27.28 followed by the Tukey-Kramer test, *p* < 0.001, **Figure 2Ba**). In neurons isolated from adolescent rats, the K^+^ current amplitude decreased to 72.2 ± 6.8% during TEA-Cl application (*p* < 0.05) and recovered to 104.9 ± 11.4% (*n* = 7, *p* < 0.01, an ANOVA *F*_(2,12)_ = 7.93 followed by the Tukey-Kramer test, *p* < 0.05, **Figure 2Bb**). In neurons of adult rats, the current amplitude decreased to 79.8 ± 7.3% during TEA-Cl application (*p* < 0.05) and recovered to 99.2 ± 2.8% after washout (*n* = 4, *p* < 0.02, an ANOVA *F*_(2,6)_ = 8.41 followed by the Tukey-Kramer test, *p* < 0.05 **Figure 2Bc**). Therefore, the unspecific blocker of K^+^ currents reversibly decreased the current amplitudes recorded in neurons that were isolated from animals of different ages.

To clarify the effect of age on BK currents, the properties of large-conductance Ca^+^^+^-dependent K^+^ single-channel currents were analyzed in young, adolescent, and adult rats.

### PROPERTIES OF SINGLE Ca^++^-DEPENDENT K^+^ CHANNEL CURRENTS IN MEDIAL PFC PYRAMIDAL NEURONS IN YOUNG, ADOLESCENT, AND ADULT RATS

Single-channel currents were recorded in the cell-attached or inside-out configuration from dispersed medial PFC pyramidal neurons. The extracellular pipette solution contained a high concentration of K^+^ ions and a Na^+^-channel blocker. The solution in the bath also contained a high concentration of K^+^ ions to keep the membrane potential close to 0 mV. Single K^+^ channel currents were recorded in pyramidal neurons isolated from young (185 channel currents), adolescent (71 channel currents), and adult (84 channel currents) rats.

**FIGURE 3:**
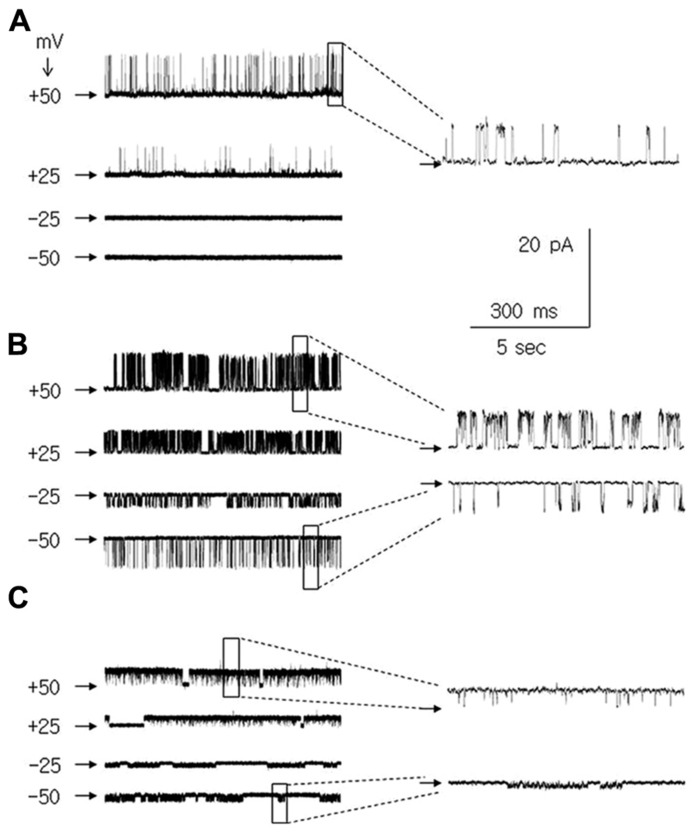
**^+^ channel currents in medial PFC pyramidal neurons recorded in the cell attached configuration at membrane potentials of +50, +25, -25, and -50 mV.** Channel current recordings at a patch potential of +50 mV and -50 mV are also shown with an expanded time base [insets to **(A–C)**]. Horizontal arrows indicate the zero current level. **(A)** Ca^+^^+^-dependent K^+^ BK-type channel currents. **(B)** Large amplitude leak channel currents. **(C)** Small amplitude leak channel currents.

**FIGURE 4:**
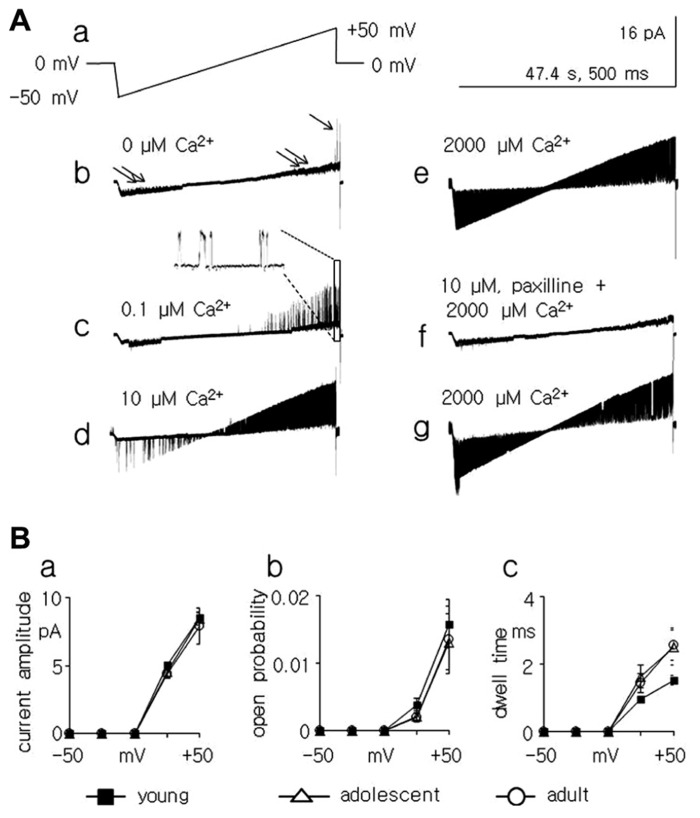
**Properties of Ca^++^-dependent K^+^ BK-type channel currents in medial PFC pyramidal neurons.** **(A)** Channel current recorded in the inside-out configuration. All results were obtained from the same patch (b–g). Two channel currents were recorded: large amplitude (1 arrow) and small amplitude (2 arrows). Only the large amplitude channel current was analyzed. A ramp membrane depolarization lasting 47.6 s from -50 to +50 mV (2.1 mV/s) was applied to evoke channel currents. This ramp was preceded by a ramp hyperpolarization lasing 1 s from 0 to -50 mV to avoid a rapid change in the membrane potential (a). Channel currents were recorded during the application, to the intracellular side of the patch membrane, of an artificial solution containing 0 μM Ca^+^^+^ (b), 0.1 μM Ca^+^^+^ (c), 10 μM Ca^+^^+^ (d), and 2000 μM Ca^+^^+^ (e). Application of 10 μM of paxilline abolished the Ca^+^^+^-dependent channel current activated by 2000 μM Ca^+^^+^ ions (f). Ca^+^^+^-dependent channel currents recovered after 8 min of wash-out (g). **(B)** Channel currents were recorded in the cell-attached configuration (as in **Figure 3A**) to analyze the channel current properties. Averaged amplitudes (a), open probabilities (b), and dwell open times (c) of BK channel currents in medial PFC pyramidal neurons at membrane potentials of -50, -25, +25, and +50 mV. Recordings were obtained from pyramidal neurons isolated from young (black boxes), adolescent (open triangles), and adult (open points) rats.

Among the single-channel currents recorded in the cell-attached configuration, large-amplitude K^+^ channel currents were found that displayed irregular dispersed openings only at positive membrane potentials (**Figure 3A** and inset). The effect of Ca^+^^+^ ions on these channels was tested in the inside-out configuration. The patch membrane potential was changed in ramp fashion from -50 to +50 mV at 2.1 mV/s (**Figure 4Aa**), and various concentrations of Ca^+^^+^ ions were applied to the intracellular side of the patch membrane. An example of the effects of the Ca^+^^+^ ions on the single-channel currents is shown in **Figure 4**. The channel displayed a few openings at a patch membrane potential close to +50 mV when the applied solution contained no Ca^+^^+^ ions (**Figure 4Ab**, single arrow). The openings changed when the concentration of Ca^+^^+^ ions on the intracellular side of the patch membrane was raised to 0.1 μM (a concentration close to that maintained in the cytoplasm at resting conditions; Clapham, 2007). The recordings displayed openings at patch membrane potentials from 0 to +50 mV, and the openings were absent at membrane potentials from 0 to -50 mV (**Figure 4Ac**). The latter results were similar to the channel currents recorded in the cell-attached configuration (**Figure 3A**). When the concentration of Ca^+^^+^ ions was raised to 10 μM, channel current openings were seen in the entire range of tested patch membrane potentials, i.e., from -50 to +50 mV (**Figure 4Ad**). The open probability of this channel current increased further when the intracellular side of the patch membrane was exposed to 2000 μM Ca^+^^+^ ions (**Figure 4Ae**). Moreover, these channel currents were completely inhibited by paxilline, which was applied to the intracellular side of the patch membrane (10 μM, **Figure 4Af**), with full recovery after 8 min of paxilline washout (**Figure 4Ag**).

We concluded that the K^+^ channel currents, which displayed only outward conductance when recorded in the cell-attached configuration, were Ca^++^ sensitive and were inhibited by paxilline are Ca^++^-dependent K^+^ BK-type channel currents (compare Benhassine and Berger, 2005, 2009; Song et al., 2010; Su et al., 2010).

The biophysical properties of BK channel currents recorded in the cell-attached configuration in medial PFC neurons isolated from animals of different ages were compared. To analyze the properties of the channel currents, data were collected at -50, -25, +25, and +50 mV patch membrane potentials. The amplitude of these channel currents increased in an outward direction when the patch membrane potential was depolarized from 0 to +50 mV (**Figure 4Ba**). A characteristic feature of these channel currents was that the open probability (**Figure 4Bb**) and dwell open time (**Figure 4Bc**) increased with the depolarization of the patch membrane. The mean amplitude, mean open probability, and mean dwell open time recorded in the cell-attached configuration at +50 mV and the mean outward conductance were not significantly different when comparing the measurements in cells obtained from young, adolescent, and adult rats (**Table 1**).

**Table 1:** Properties of Ca^++^-dependent K^+^ channel currents in pyramidal neurons isolated from young, adolescent, and adult rats.

Age	Young	Adolescent	Adult	
*n*	72	8	19	
Conductance (pS)	146.59 ± 17.90	158.81 ± 20.36	160.25 ± 45.99	One-way ANOVA, *p* = 0.7122
Amplitude (pA) at +50 mV	8.56 ± 0.37	8.50 ± 0.48	7.94 ± 0.52	One-way ANOVA, *p* = 0.6945
Open probability at +50 mV	0.015 ± 0.0036	0.013 ± 0.0042	0.013 ± 0.0049	Kruskal–Wallis, *P* = 0.079
Dwell open time (ms) at +50 mV	1.51 ± 0.14	2.51 ± 0.56	2.00 ± 0.45	Kruskal–Wallis, *p* = 0.7657

In addition to the BK channel currents, two types of K^+^ leak channel currents with large (**Figure 3B**) and small conductance (**Figure 3C**) were found in neurons isolated from young, adolescent, and adult rats. When the recordings were performed in the cell-attached configuration, they displayed openings at positive and negative membrane potentials. The outward conductances of large leak channel currents were not significantly different in the pyramidal neurons obtained from young, adolescent, and adult rats. Furthermore, the inward conductances of the large conductance leak channel currents were not significantly different in the neurons from the three groups of animals (**Table 2**). The outward conductances of the small leak channel currents in the pyramidal neurons were also not different across ages. The inward conductances of small leak channel currents were also not different (**Table 2**).

**Table 2:** Properties of large and small leak channel currents recorded from pyramidal neurons isolated from young, adolescent, and adult rats.

Leak channel currents		Young	Adolescent	Adult	One-way ANOVA
Large conductance (pS)		*n* = 72	*n* = 46	*n* = 50	
	Outward	143.50 ± 6.52	135.7 ± 6.35	144.12 ± 8.02	*p* = 0.9458
	Inward	160.25 ± 5.99	149.2 ± 5.67	163.40 ± 3.38	*p* = 0.4341
Small conductance leak		*n* = 31	*n* = 13	*n* = 12	[-1pc]
channel currents (pS)	Outward	37.21 ± 3.56	23.97 ± 6.39	41.13 ± 9.67	*p* = 0.9133
	Inward	40.55 ± 4.83	27.51 ± 4.47	32.08 ± 7.70	*p* = 0.2894

It is likely that a single type of BK channel was recorded and analyzed in our study; the currents displayed very low open probability and had nearly identical amplitude. In addition, when the intracellular side of the patch was exposed to Ca^+^^+^ ions, the maximum current activation did not lead to double openings (two or more levels of openings, **Figure 3A** and **4**; **Table 1**; compare Horn, 1991; Chen and Johnston, 2004; Sakmann and Neher, 2009).

**FIGURE 5:**
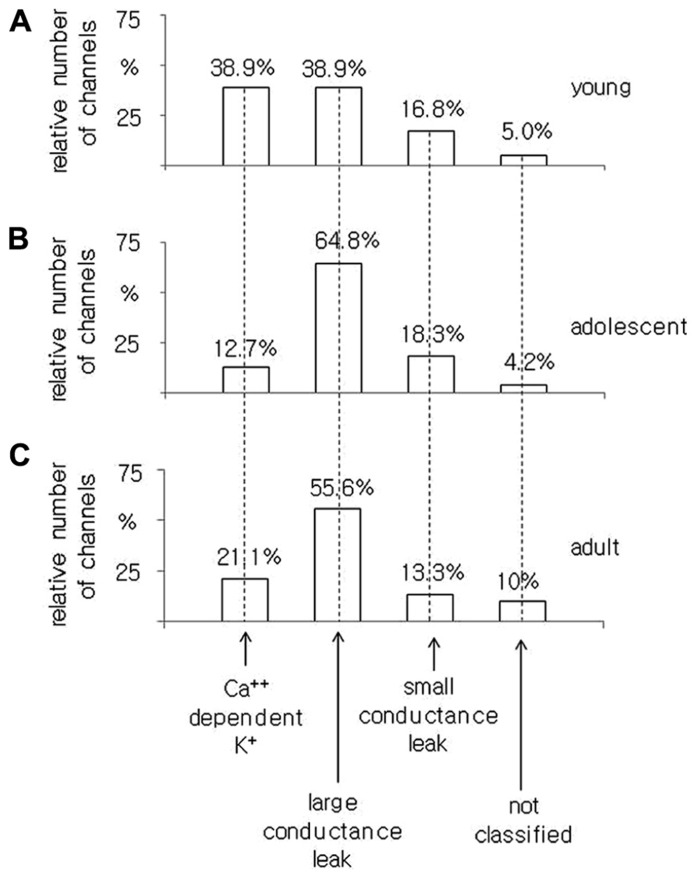
**Relative number of Ca^+^^+^-dependent K^+^ BK-type, large conductance, small conductance leak and unclassified channel currents recorded in the cell-attached configuration from pyramidal neurons isolated from young **(A)**, adolescent **(B)**, and adult **(C)** rats**.

In young rats, of the 185 channel currents recorded from the pyramidal neurons, 38.9% were BK channel currents, and 38.9 and 16.8% were large and small conductance leak channel currents, respectively. A total of 5% of the channel currents were not included in any groups (**Figure 5A**).

In the pyramidal neurons obtained from adolescent animals, 71 channel currents were recorded. There were 12.7, 64.8, and 18.3% channel currents with the properties of BK, large conductance and small conductance leak channel currents, respectively. A total of 4.2% of the channel currents were not classified (**Figure 5B**).

In the pyramidal neurons dissected from adult rats, we found that 21.1, 55.6, and 13.3% were BK, large and small conductance leak channel currents, respectively. A total of 10% of the channel currents were not included in any group out of all 90 channel currents tested, **Figure 5C**.

The obtained results indicate that the smallest proportion of BK channel currents was found in the pyramidal neurons obtained from adolescent rats as opposed to the pyramidal neurons isolated from young and adult animals.

### EFFECT OF PAXILLINE ON THE ACTION POTENTIAL HALF-WIDTH IN MEDIAL PFC PYRAMIDAL NEURONS IN YOUNG, ADOLESCENT, AND ADULT RATS

**FIGURE 6:**
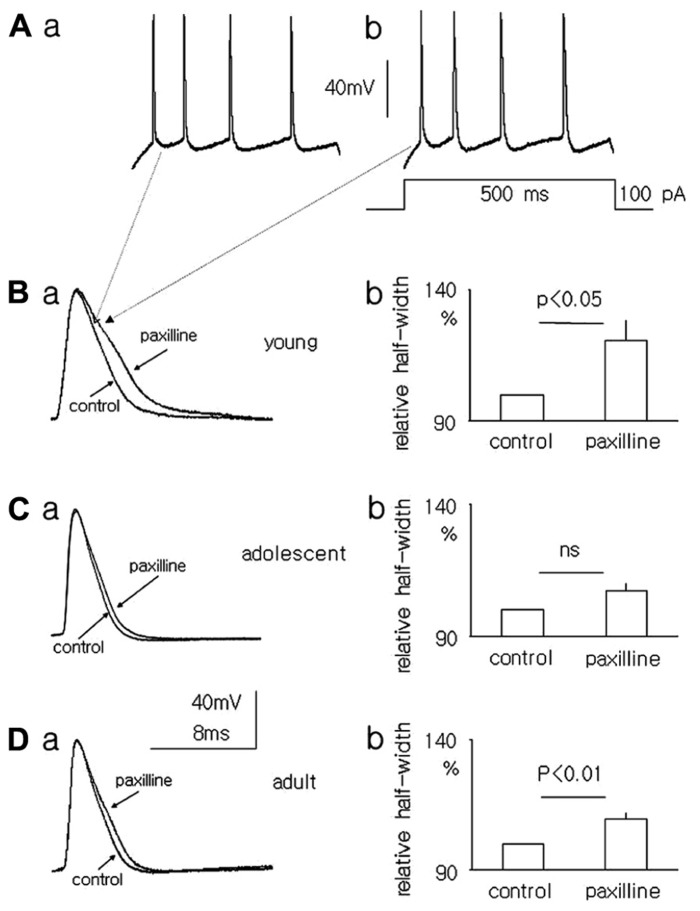
**Effect of paxilline (10 μM) on the action potential half-width in pyramidal neurons in slices from the medial PFC.** Example of action potentials evoked by a 100-pA voltage step lasting 500 ms [inset to **(Ab)**] in pyramidal neurons isolated from the medial PFC of young rats before **(Aa)** and after a 5-min application of paxilline **(Ab)**. Overlapping action potentials recorded before (control) and after paxilline (paxilline) application in pyramidal neurons isolated from young **(Ba)**, adolescent **(Ca)**, and adult **(Da)** rats. Relative averaged half-width of the action potentials recorded in pyramidal neurons isolated from young **(Bb)**, adolescent **(Cb)**, and adult **(Db)** rats.

The resting membrane potentials recorded in the whole-cell configuration from layer V medial PFC pyramidal neurons in the slices of young, adolescent and adult rats were -65.4 ± 2.0 mV (*n* = 6), -66.3 ± 0.85 mV (*n* = 11), and -67.3 ± 1.55 mV (*n* = 8), respectively. To evoke action potentials (**Figures 6Aa,b**), depolarizing current steps in 10 pA increments from 10 to 120 pA lasting 500 ms were applied once every 4 s. The half-width of the first action potential in the train (in traces containing 3–4 action potentials) was measured before and after 5 min of paxilline (10 μM) application to the bath. After a 5-min application of paxilline the action potential half-width significantly increased to 117.0 ± 6.5% relative to 100% for the control (*n* = 6, paired *T*-test, *p* < 0.05, **Figures 6Ba,b**) in the pyramidal neurons of young rats. In adolescent rats, the action potential half-width also increased but not significantly (107.1 ± 3.33 vs. 100% control, *n* = 11, paired *T*-test, *p* > 0.05, **Figures 6Ca,b**). In adult rats, the action potential half-width increased significantly to 109.5 ± 2.7% from 100% for the control (*n* = 8, paired *T*-test, *p* < 0.01, **Figures 6Da,b**).

## DISCUSSION

The aim of this study was to describe the properties and functional expression of Ca^+^^+^-dependent K^+^ BK-type currents in the pyramidal neurons of the medial PFC in young, adult, and adolescent rats. The obtained results indicate that the BK current availability is lower in neurons isolated from adolescent animals than in neurons obtained from young and adult rats. Twenty-day-old rats, which are at the weaning age, were included in the group of young animals. It is assumed that 40-day-old rats are in the middle of the periadolescent phase (Spear, 2000; McCutcheon and Marinelli, 2009). During periadolescence, abrupt changes in the prefrontal cortex occur with respect to the expression and properties of different ion channels and synaptic transmission (Zhu, 2000; Andersen, 2003; Zhang, 2004; Tseng and O’Donnell, 2007; Brenhouse et al., 2008; Wang and Gao, 2009, 2010; Heng et al., 2011). Sixty-day-old rats are also termed young-adult rats (Spear, 2000; McCutcheon and Marinelli, 2009). This age was preferred because long-lasting recordings from older animals from the dispersed neurons and neurons in slices were difficult to obtain.

### BK CURRENTS RECORDED FROM PYRAMIDAL NEURONS IN THE WHOLE-CELL CONFIGURATION FOR SAMPLES OBTAINED FROM YOUNG, ADOLESCENT, AND ADULT RATS

After the blockade of Na^+^ and Ca^+^^+^ channels, K^+^ currents were recorded from dispersed pyramidal neurons in whole-cell configuration. The membrane potential was close to 0 mV in “symmetrical” K^+^ solutions; this potential inactivated voltage-dependent and fast-inactivating K^+^ currents (type K_A_, K_D_, Rola et al., 2003; Szulczyk and Szulczyk, 2003). The concentration of Ca^+^^+^ ions in the pipette (intracellular) solution was raised to 10 μM to activate Ca^+^^+^-dependent K^+^ currents and to lower their voltage threshold (Sun et al., 1999; Benhassine and Berger, 2005; Song et al., 2010 and this study). In these findings, the fraction of whole-cell current that was blocked by paxilline was primarily from the BK-type Ca^+^^+^-dependent K^+^ current (Li and Cheung, 1999; Benhassine and Berger, 2009).

It was found that paxilline partially inhibited the whole-cell outward K^+^ current in neurons isolated from young and adult animals. Paxilline binds to the intracellular side of BK channels. Because it is lipophilic, paxilline can enter the cytoplasm and block the BK channel current (Knaus et al., 1994). There was no recovery of the whole-cell K^+^ current during prolonged washout. We hypothesized that prolonged current inhibition was caused by paxilline trapped in the cell. The lack of current recovery observed in young and adult animals raised the suspicion that the current inhibition was caused by K^+^ current run-down despite the precautions taken to eliminate it. To exclude this possibility, we tested the effect of the reversible K^+^ channel blocker, TEA-Cl, at a concentration 1.5 mM on the currents from pyramidal neurons. The whole-cell current was inhibited during TEA-Cl application and fully recovered after TEA-Cl wash-out in neurons isolated from young, adolescent, and adult animals. As run-down was not observed after TEA-Cl washout, we concluded that run-down was not responsible for the decrease in current amplitude during paxilline application and washout in young and adult rats.

TEA-Cl inhibits 20–30% of the total whole-cell K^+^ current in young, adolescent, and adult rats. The whole-cell currents recorded in this study were composed of several K^+^ channel currents expressed in the soma, apical, and basal dendrites and, sometimes, axon remnants (Figure 1 in Witkowski and Szulczyk, 2006). These channels include TREK (Talley et al., 2001 and this study), BK (Benhassine and Berger, 2005 and this study), K_DR_ (delayed rectifier potassium currents; Dong and White, 2003; Chen and Johnston, 2004), GIRK (G-protein-coupled inward rectifying K^+^ channel current; Witkowski et al., 2008), and IRK (inward rectifying K^+^ currents; Dong et al., 2004) channels, among others. TREK (Liu et al., 2007), BK (Sun et al., 1999), K_DR_ (Rola et al., 2003), GIRK (Yamada et al., 1998), and IRK (Hibino et al., 2010) channels are all inhibited by TEA-Cl. Therefore, it is not surprising that the whole-cell K^+^ current is partially inhibited by TEA-Cl in pyramidal neurons isolated from young, adolescent and adult animals.

We find that application of paxilline evokes an insignificant increase in the whole-cell K^+^ current in dispersed pyramidal neurons of adolescent rats. It was demonstrated by others that paxilline exerts an additional action in cells; it may inhibit the sarco/endoplasmic reticulum Ca^+^^+^ ATPase (SERCA). This leads to decreased Ca^+^^+^ uptake by the sarcoplasmatic reticulum, thereby increasing the Ca^+^^+^ concentration in the cell. In cells there is a balance between Ca^+^^+^ uptake by the sarcoplasmatic reticulum (by SERCA) and Ca^+^^+^-induced Ca^+^^+^ release from the sarcoplasmatic reticulum (Albrecht et al., 2002). The application of paxilline may inhibit SERCA, leading to an imbalance between Ca^+^^+^ uptake and Ca^+^^+^ release from the sarcoplasmatic reticulum. This suggests that during paxilline application, the concentration of Ca^+^^+^ ions in the cell may increase compared to control conditions. Ca^+^^+^-dependent SK channels are expressed in the dendrites of pyramidal neurons (Gu et al., 2008; Faber, 2010). Thus, the increase in Ca^+^^+^ ion concentration due to the inhibition of Ca^+^^+^ uptake may generate additional small (and statistically insignificant) outward SK currents during depolarizing voltage steps. This effect is not visible in pyramidal neurons isolated from young and adult rats; paxilline markedly inhibits the BK channels that are predominant in these neurons.

The recordings performed in whole-cell configuration suggest that paxilline-sensitive BK-type K^+^ currents dominate in the pyramidal neurons of young and adult rats. Paxilline does not inhibit the whole-cell current in adolescent rats, suggesting that this current is of less importance in this group.

### PROPERTIES OF BK CHANNEL CURRENTS RECORDED FROM PYRAMIDAL NEURONS OBTAINED FROM YOUNG, ADOLESCENT, AND ADULT RATS

Ca^+^^+^-dependent K^+^ channel currents fall into three general populations: BK-type channels that have a large conductance (>100 pS), SK-type channels that have a low conductance (<20 pS), and intermediate conductance-type channels (IK, 20–50 pS, Sah and Faber, 2002). Both BK (Benhassine and Berger, 2005) and SK (Faber, 2010) channels are present in cortical pyramidal neurons. The intermediate conductance channels are not found in any neurons (Ishii et al., 1997). The subtypes of Ca^+^^+^-dependent K^+^ channel currents differ in voltage sensitivity. BK currents are sensitive to voltage, whereas SK channels are voltage independent (Faber, 2010).

The channel currents described in this study, which were paxilline-, Ca^+^^+^-, and voltage-dependent and had an outward conductance of ~150 pS, were most likely Ca^+^^+^-dependent K^+^ BK-type channel currents. These channels, when recorded in the cell-attached configuration, displayed only outward conductance. This finding is in agreement with earlier reports. The concentration of Ca^+^^+^ ions in the cytoplasm is approximately 100–200 nM in physiological conditions (Silver and Erecinska, 1990; Nakamura et al., 1999). At this concentration of Ca^+^^+^ ions and in “symmetrical” K^+^ ion solutions, the voltage threshold for the BK channel current is close to 0 mV or higher, and therefore, only an outward single BK channel current can be recorded (Sun et al., 1999; Benhassine and Berger, 2005; Ha et al., 2006; Song et al., 2010, see also the experiments of this study).

When recordings of the BK channel currents were performed in the cell-attached configuration, the conductance, amplitude, open probability, and dwell open times at the tested patch membrane potentials did not differ across animals of different ages. Therefore, one may conclude that the lower amplitude of the BK current recorded in the whole-cell configuration, as seen in the pyramidal neurons of adolescent animals relative to the pyramidal neurons of young and adult animals, did not depend on the age-dependent biophysical properties of single BK channel currents.

In our study, we also found K^+^ channel currents whose amplitudes changed in an outward and inward direction during cell depolarization and hyperpolarization, respectively, when recorded in the cell-attached configuration in a symmetrical K^+^ solution. Therefore, these channel currents had properties of leak (background) channel currents (Lotshaw, 2007; Enyedi and Czirják, 2010). TREK channels were found in medial PFC pyramidal neurons (Talley et al., 2001; Aller and Wisden, 2008). These channels have a large conductance and have properties of leak channel currents (Maingret et al., 1999; Bang et al., 2000; Han et al., 2003). Small conductance leak channel currents were also found in the forebrain; these channels had properties of TASK-type channels (Talley et al., 2001; Aller and Wisden, 2008; Enyedi and Czirják, 2010).

It was found that 38.9% of single, non-inactivating K^+^ channel currents recorded in the medial PFC of young animals showed the properties of BK channels. Among all of the channels recorded in the neurons obtained from adolescent animals, 12.7% had properties of BK channels. In the pyramidal neurons of adult animals, 21.1% were BK channels. This result suggests that BK channels are expressed as a smaller proportion of all K^+^ channels in the pyramidal neurons of adolescents relative to those in young and adult animals.

### EFFECT OF PAXILLINE ON ACTION POTENTIALS RECORDED IN PYRAMIDAL NEURONS ISOLATED FROM YOUNG, ADOLESCENT, AND ADULT RATS

The outward Ca^+^^+^-dependent K^+^ BK-type current is involved in action potential repolarization (Storm, 1987, 1988, 1990; Sah and Faber, 2002; Faber and Sah, 2003). Because paxilline is a selective blocker of BK currents (Li and Cheung, 1999; Benhassine and Berger, 2009), its application diminishes outward K^+^ currents, slows down action potential repolarization and increases the action potential half-width (Faber and Sah, 2003; Benhassine and Berger, 2005, 2009; Song et al., 2010; Su et al., 2010).

Different collections of ion channels might be activated and analyzed when the recordings were performed from dispersed neurons or from cells in slices. Whole-cell K^+^ currents recorded in dispersed neurons were the result of ion channel activation which were expressed in the soma and residual neuron processes. Single channel currents from dispersed neurons were recorded from channels expressed only in the soma. Action potential recordings were the result of coordinated activation of ion channels expressed in the soma and processes of the neurons. There are indications that the channel properties and expression might differ in various compartments of the neuron (Magee and Johnston, 1995). Despite these potential differences in K^+^ channel current properties in different experimental conditions, the outcome of the experiments with action potential recordings is consistent with the results obtained during K^+^ current recordings. The results of our study indicate that the action potential half-width did not significantly change during paxilline application in the neurons of adolescent animals and was prolonged in the neurons isolated from young and adult animals.

There was also a small, insignificant increase in the action potential half-width in adolescent animals that was compatible with the presence of residual BK channels in the soma of pyramidal neurons.

This result supports the finding that functional expression of BK channel currents is lower in adolescent animals relative to that in animals of other ages.

### FUNCTIONAL SIGNIFICANCE

Opening of voltage-dependent Ca^+^^+^ channels depends on the action potential half-width. Broadening of the action potential, due to lower availability of BK channels, may lead to prolonged opening of Ca^+^^+^ channels and loading of the cell with Ca^+^^+^ ions. Ca^+^^+^ ions serve as a second messenger that affects multiple cellular functions (Berridge, 1998). Ca^+^^+^ ions may trigger development of dendritic spines during adolescence (Lemieux et al., 2012). Ca^+^^+^ ions also prolong medial PFC pyramidal neuron depolarization during adolescence (Heng et al., 2011). In addition, lower expression of BK channels should increase dendritic excitability in neocortical pyramidal neurons (Benhassine and Berger, 2009). Therefore, lower availability of BK channels during adolescence may facilitate pyramidal neuron activity during this phase of development.

## Conflict of Interest Statement

The authors declare that the research was conducted in the absence of any commercial or financial relationships that could be construed as a potential conflict of interest.
